# Nature and nurture: understanding phenotypic variation in inborn errors of immunity

**DOI:** 10.3389/fcimb.2023.1183142

**Published:** 2023-09-14

**Authors:** Morgan Similuk, Taco Kuijpers

**Affiliations:** ^1^Centralized Sequencing Program, Division of Intramural Research, National Institute of Allergy and Infectious Diseases, National Institutes of Health, Bethesda, MD, United States; ^2^Department of Pediatric Immunology, Rheumatology and Infectious Diseases, Emma Children’s Hospital, Amsterdam University Medical Center, University of Amsterdam, Amsterdam, Netherlands

**Keywords:** inborn error of immunity, genetics, environment, time, penetrance, phenotypic variation

## Abstract

The overall disease burden of pediatric infection is high, with widely varying clinical outcomes including death. Among the most vulnerable children, those with inborn errors of immunity, reduced penetrance and variable expressivity are common but poorly understood. There are several genetic mechanisms that influence phenotypic variation in inborn errors of immunity, as well as a body of knowledge on environmental influences and specific pathogen triggers. Critically, recent advances are illuminating novel nuances for fundamental concepts on disease penetrance, as well as raising new areas of inquiry. The last few decades have seen the identification of almost 500 causes of inborn errors of immunity, as well as major advancements in our ability to characterize somatic events, the microbiome, and genotypes across large populations. The progress has not been linear, and yet, these developments have accumulated into an enhanced ability to diagnose and treat inborn errors of immunity, in some cases with precision therapy. Nonetheless, many questions remain regarding the genetic and environmental contributions to phenotypic variation both within and among families. The purpose of this review is to provide an updated summary of key concepts in genetic and environmental contributions to phenotypic variation within inborn errors of immunity, conceptualized as including dynamic, reciprocal interplay among factors unfolding across the key dimension of time. The associated findings, potential gaps, and implications for research are discussed in turn for each major influencing factor. The substantial challenge ahead will be to organize and integrate information in such a way that accommodates the heterogeneity within inborn errors of immunity to arrive at a more comprehensive and accurate understanding of how the immune system operates in health and disease. And, crucially, to translate this understanding into improved patient care for the millions at risk for serious infection and other immune-related morbidity.

## 1 Introduction

Clinical heterogeneity is common among thousands of described Mendelian disorders (Chong et al., 2015; Posey et al., 2019). Likewise, for inborn errors of immunity (IEIs) reduced penetrance and variable expressivity are the rule, not the exception (Gruber and Bogunovic, 2020; Aksentijevich and Schnappauf, 2021). Even the severe combined immune deficiencies, the most paradigmatic of examples, are not uniformly apparent in infancy (Dvorak et al., 2023). Moreover, clinical presentations of IEIs are particularly reliant on environmental triggers, namely, exposure to pathogens, to begin to manifest most symptoms (Casanova and Abel, 2021). The implications that this variability and uncertainty has for families – medically, emotionally, and socially – can be vexing both for the affected individuals and their care teams.

The issue of penetrance is not only a pervasive phenomenon among IEI and other Mendelian diseases, but it is also among the most fundamental questions in human genetics: what is the probability of phenotypic expression given the genotype? Historically, efforts to dissect genotype-phenotype correlation have been largely dichotomized between rare variants of large effect and common variants of small effect on risk for disease, although this has almost certainly obscured the true nature of the underlying biology with disease risk variants being distributed across a continuum of frequencies and effect sizes (Antonarakis et al., 2010; Claussnitzer et al., 2020). Critically, recent advances are illuminating additional nuance for fundamental concepts in understanding penetrance, as well as raising new areas of inquiry. For example, genome-wide analyses in large, population-based biobanks have repeatedly confirmed the suspected inflation of Mendelian disease penetrance estimates when such estimates are clinically ascertained through proband analysis or family studies and have enabled a more accurate, more comprehensive understanding of the relationship between genotype and phenotype for select diseases (Ranola et al., 2019; Tuke et al., 2019; Wright et al., 2019). Concurrently, advances in sequencing have enabled the detection of somatic variants, microbiome composition, and other relevant factors, to an extent not previously possible. If the prior generations of immunologists grappled with the bottlenecks of variant discovery and disease association, the current era will be one defined by efforts to understand the mechanisms by which the full spectrum of genetic variation influences biology and to translate that understanding into clinical care (Lappalainen and MacArthur, 2021). It is a formidable challenge, yet demonstrably tractable, with evidence of progress year after year.

**Figure 1:**
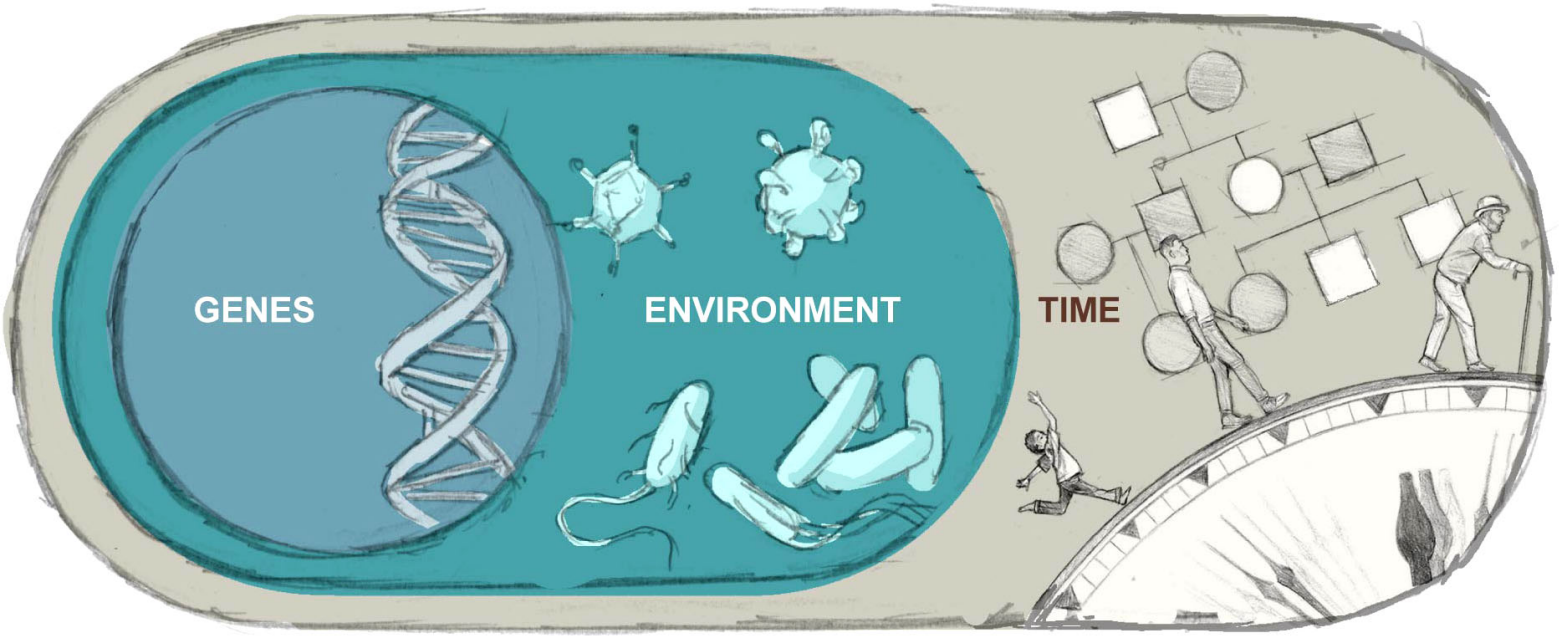
Illustrates the complex interplay among an individual’s genetic and environmental contributions to health and disease over time.

As such, the purpose of this review is to provide an updated summary of key concepts in genetic and environmental contributions to phenotypic variation within IEI, conceptualized as including dynamic, reciprocal interplay among factors (**Figure 1**). The associated controversies, potential gaps, and implications for research are discussed throughout, as are the connections to patient care.

## 2 Body

### 2.1 Genetics

We review IEI-specific examples of differential variant impact on phenotype, a foundational concept in genetics, followed by contemporary discussions of mosaicism, allelic bias, and multi-locus genetic variation.

#### 2.1.1 Differential variant impact

**Figure 2:**
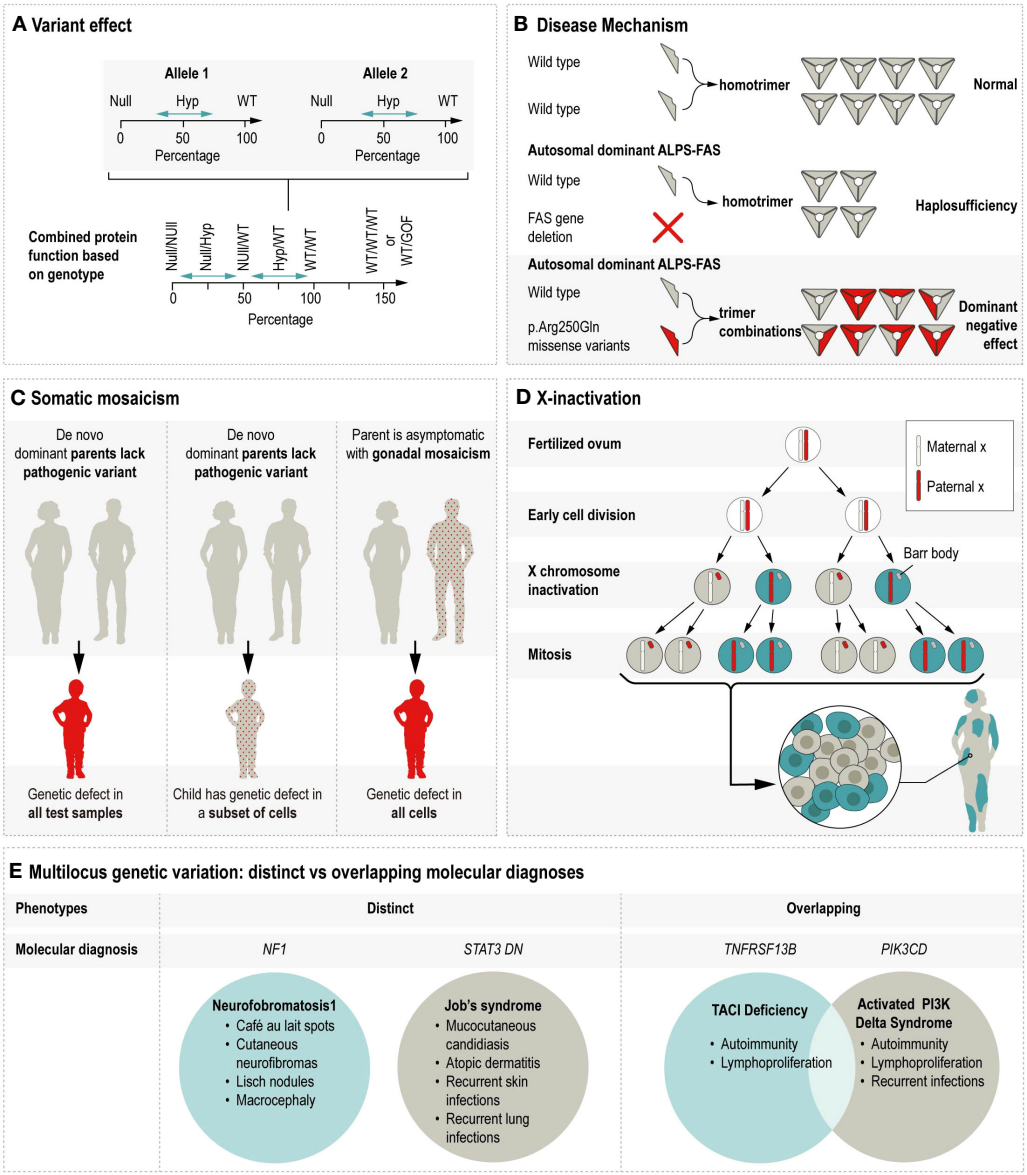
Illustrates the multiple mechanisms by which genetic variation contributes to phenotypic variability. Hyp = Hypomorphic, WT = Wildtype, GOF = Gain of function, ALPS-FAS = Autoimmune lymphoproliferative syndrome due to a defect in *FAS*, DN = Dominant negative. **(A)** Variant effect on protein function is shown as the combined impact of allele 1 and allele 2. For example, the combined protein function resulting from two null alleles would be less than the protein function resulting from hypomorphic alleles with some residual function. In some cases, triplosensitivity (WT/WT/WT) or alleles with GOF effects can produce an increased level of protein function. (Adapted from Zschocke et al., 2023.) **(B)** The disease mechanisms of halploinsufficiency and dominant negative are contrasted using the example of monoallelic ALPS-FAS. Ordinarily, the two wildtype proteins homotrimerize to create the transmembrane protein which initiates caspase cascade upon activation. In cases where one allele is affected by a FAS gene deletion, the single wildtype allele produces half the normal amount of homotrimers, leading to ALPS via haploinsufficiency. In cases where one allele is affected by a pathogenic missense variant, the mutant protein is incorporated in 7 of 8 possible configurations, thus leading to ALPS via dominant negative action. **(C)** Somatic mosaicism’s impact on autosomal dominant inheritance varies by the timing of the mutational event. In the left panel, the parents lack the pathogenic variant in all their cells, the pathogenic variant arises early in embryonic development, and is present in all tested samples obtained from the affected child. The middle panel is similar, except that in this example the pathogenic variant arises later in development, such that the child is still symptomatic but that the genetic defect is present in some but not all of the tested cells types. In the right panel, the pathogenic variant is present at a low frequency in an unaffected parent and subsequently passed on to the affected child who has the pathogenic variant in all of their cells. **(D)** X-inactivation creates cellular uniallelic expression in XX females through the inactivation of one X chromosomes early in embryonic development. As a result, XX females mosaically express X chromosomes throughout their body. **(E)** Patients with multiple molecular diagnoses generally either have molecular diagnoses reflecting distinct or overlapping disorders. For example, the disorders caused by defects in *NF1* and dominant negative STAT3 are distinct such that the respective symptoms can be reasonably attributed to either the *NF1* or *STAT3* defect. In contrast, the symptoms associated with defects in *TNFRSF13B* and *PIK3CD* are largely overlapping and not possible to distinguish.

Discerning the molecular consequence of a variant is a critical clue in anticipating its potential cellular and ultimate phenotypic impact. There are many related terms used to describe aspects of variant effects, including the effect on the protein sequence (e.g., missense, frameshift, stop gained, synonymous, etc.), the genetic mechanism of disease (e.g., haploinsufficiency, dominant negative), and protein function (e.g., gain-of-function or GoF; loss-of-function or LoF) (Nussbaum et al., 2015; Zschocke et al., 2023). GoF typically refers to the enhancement of the affected protein’s activity, and occasionally refers to a novel or neomorphic function, as is the case in a recently described *CEBPE*-related autoinflammatory disorder in which the defect lead to enhanced chromatic occupancy and ultimately dysregulated signaling (Göös et al., 2019). See **supplemental table** for description of GOF IEI. The term LoF is sometimes replaced with more specific descriptors such as null, meaning eliminated activity, or hypomorphic, meaning diminished activity or presence of the encoded protein (Ott et al., 2023). See **Figure 2A** for illustration.

Identifying the diminishment or gain of protein function is a necessary first step in understanding disease mechanisms and is dependent on the variant’s impact on the biological pathway(s) associated with the protein(s) encoded by the gene. The discovery of new mechanisms of disease among known IEI-associated genes accounted for multiple additions to the most recent International Union of Immunological Sciences (IUIS) categorizations of IEI (e.g., *STAT2* LoF vs GoF; *IKZF1* LoF vs GoF) (Tangye et al., 2021; Tangye et al., 2022). For the discussion at hand, it should be underscored that such differences in protein effect do not contribute to intrafamilial variation in the context of a familial variant.

Indeed, we are only beginning to understand the patterns associated with gain or loss of function at the level of the variant, gene, and protein. A recent comprehensive analysis found that GoF variants are enriched in essential genes, for autosomal dominant inheritance, and localized in protein binding and protein interacting domains; whereas LoF variants are enriched for protein-truncating variants in protein core regions where variants may disrupt the stability and folding of the protein (Sevim Bayrak et al., 2021). Such insights extend our knowledge of the properties associated with LoF or GoF and contribute to a growing ability to accurately predict variant impact.

This progress notwithstanding, GoF vs LoF effects can be very difficult to discern even with direct functional data, particularly when the underlying biology is complex, as highlighted in the case of a *CARD11* variant with pathogenic features of both GoF and LoF effects (Desjardins et al., 2018). As such, understanding phenotypic variation in IEI requires further development of *in silico* tools to prioritize candidate variants and complement the strong history of experimental work in clinical immunology.

Mendelian susceptibility to mycobacterial disease (MSMD) provides an illuminating clinical example of differential variant impact on loss of immune function and subsequent phenotype (Bustamante et al., 2014). MSMD is characterized by predisposition to disease from poorly virulent mycobacteria (e.g., Bacillus Calmette–Guérin (BCG) vaccines and environmental mycobacterial) in otherwise healthy individuals and can be caused by defects in multiple genes (Bustamante et al., 2014; Bustamante, 2020). As one example, multiple defects affecting *IFNGR1* cause MSMD. *IFNGR1* encodes the IFN-γ receptor-ligand binding chain and thus, when mutated interferon and related signaling are dysregulated. Within *IFNGR1*-related MSMD, biallelic, null variants cause the most severe phenotype of disseminated, life-threatening infections with nearly complete penetrance in childhood. In these cases, the nonsense or frameshift variants create a premature stop codon and prevent the expression of IFNGR1 at the cell surface (Holland et al., 1998; Filipe-Santos et al., 2006). In contrast, both biallelic, hypomorphic variants and heterozygous monoallelic, autosomal dominant (partial) IFNGR1 deficiency have a milder clinical phenotype and reduced, age-related penetrance (Jouanguy et al., 1997; Jouanguy et al., 1999). Management of the *IFNGR1*-related MSMD clinical spectrum varies by severity, with poor prognosis in the former case and infrequent need for prophylactic antibiotics or hematopoietic stem cell transplantation (HSCT) in the latter cases (Holland et al., 1998; Filipe-Santos et al., 2006). Thus, differential variant impact is highly consequential for MSMD.

Relatedly, the clinical spectrum arising from RAG (“recombination activating genes”) deficiency provides another example of the effect of variant type on immune function and one that contains both marked inter- and intra-familial variation in clinical manifestations (Bosticardo et al., 2021).

As an additional consideration for variant impact on phenotypic variation, protein function is not equally disrupted by all pathogenic variation along the length of a gene. Understanding the function of a protein’s domains can provide insight into a given variant’s potential consequences. The different molecular and clinical consequences of FAS defects provide one example. The gene *FAS* encodes a cell surface receptor involved in apoptosis and is critical in maintaining cellular homeostasis (Fisher et al., 1995). Defects in FAS cause autoimmune lymphoproliferative syndrome (ALPS), which is characterized by autoimmunity, particularly against components of the blood; lymphoproliferation including lymphadenopathy; and an increased risk for lymphoma (Price et al., 2014). Heterozygous pathogenic variants in the *FAS* “death domain” interfere with the formation of the homotrimeric receptor. In the presence of a heterozygous mutation, seven out of eight homotrimers will contain at least one defective copy of the FAS protein, thus, diminishing effective signaling through negative interference (Kuehn et al., 2011).

For variants outside of this domain or *FAS* deletions, the mechanism is haploinsufficiency, whereby the total amount of FAS protein generated is below the threshold for apoptosis induction (Hsu et al., 2012; Jevtich et al., 2022). Critically, these different mechanisms may influence penetrance, meaning the likelihood that a genetic defect will cause symptoms. The penetrance of ALPS due to negative interferences was reported as up to 60% (Price et al., 2014), whereas haploinsufficiency for FAS is associated with a penetrance estimate of 40% (Kuehn et al., 2011). This is a prime example of distinguishing dominant negative biological effects, which can be distinguished from that of haploinsufficiency. See **Figure 2B** for illustration. Lastly, apart from the residual surface expression, biallelic inheritance of FAS defects results in a more severe, more penetrant ‘FAS-null’ phenotype (Rieux-Laucat et al., 1995).

*AIRE*-related autoimmune polyendocrinopathy-candidiasis-ectodermal dystrophy (APECED) also exhibits phenotypic variation based on the location of the pathogenic variant and subsequent genetic mechanism. AIRE (autoimmune regulator) is a gene expressed in the thymus and involved in establishing immune tolerance. Defects results in the peripheral escape of self-reactive T lymphocytes and autoantibodies. APECED is classically characterized by chronic mucocutaneous candidiasis, hypothyroidism, and adrenal insufficiency (Ferré et al., 2021). While biallelic LoF variants are the most common cause of APECED, dominant-negative variants within the SAND and PHD1 domains of the *AIRE* gene have been reported as a cause of APECED. The dominant-negative variants are associated with reduced penetrance, milder symptoms, and fewer autoimmune manifestations compared to biallelic LoF variants (Ferré et al., 2021), similar as observed with the aforementioned *STAT1* biallelic or dominant-negative variants. Genotype and phenotype correlation, particularly by distinct disease mechanisms is a very active area of research and there are many additional and recent examples including disorders caused by defects in *WAS*, *RAC2*, *IKZF1*, and *PSTPIP1*, among others, with a distinct phenotype when comparing the LoF and GoF variants (Holzinger et al., 2015; Masters et al., 2016; Kuehn et al., 2021; Hoshino et al., 2022; Hsu, 2023).

Lastly, future work will likely continue to provide illustrative examples of differential variant impact for rare IEI, as well as identify additional disease-associated allelic series across the spectrum of frequency and effect size. A current example of allelic series in IEI involves the gene *TYK2* (tyrosine kinase 2), which mediates immune and inflammatory signaling (Casanova and Abel, 2022). Rare biallelic variants in *TYK2* predispose to infection by intracellular bacteria or viruses, while at the same time, common *TYK2* variants are associated with inflammatory and autoimmune disease (Li et al., 2020; Casanova and Abel, 2022). Similarly, rare variants causing haploinsufficiency of CTLA4 (cytotoxic t-lymphocyte associated protein 4) impair Treg function, leading to a syndrome of hypogammaglobulinemia, lymphoproliferation, and autoimmune cytopenia with respiratory, gastrointestinal, and/or neurological features (Schwab et al., 2018). In contrast, select common variants in *CTLA4* have been reported in association with complex autoimmune disorders such as rheumatoid arthritis and hypothyroidism (Schwab et al., 2018; Buniello et al., 2019). The same applies to LRBA as CTLA4, being in the same pathway of Treg function and other autoinflammatory conditions such as inflammatory bowel disease, with a potential for therapeutic approaches to improve their regulatory cell function (Gettler et al., 2021). Only by better understanding the full spectrum of how genetic variation influences biology will we arrive at a comprehensive view of genotype-phenotype correlations with translational importance for patient diagnosis, prognosis, and treatment.

#### 2.1.2 Mosaicism

Genetic mosaicism is emerging as an important contributor to many disorders, including IEI molecular diagnostics as well as more complex disease processes. Somatic mosaicism refers to post-zygotic changes to the inherited, germline DNA sequence, which are present at variable allele fractions and distributions among cells throughout the body. Germline mosaicism refers to the heterogenous variant status of the diploid germ cell precursors in one’s gonads: some cells have the variant and some do not (Biesecker and Spinner, 2013). By one estimate, approximately 4% of apparently *de novo* pathogenic variants are present at a frequency >1% in parental blood, with important implications for recurrence risk counseling (Rahbari et al., 2016). See **Figure 2C** for illustration.

At one end of the spectrum, high-impact somatic events occurring early in post-zygotic development can lead to IEIs that are phenotypically and genotypically indistinguishable from disorders caused by germline variants, as shown in two cases of *STAT5B*-related allergic disease and autoimmunity caused by the same somatic variant arising in a hematopoietic progenitor (Ma et al., 2017). The observed frequency of somatic variants leading to ‘monogenic’ IEI is dependent on cohort selection and technologies used, as low allele fraction somatic variants are beyond the limits of detection for common next-generation sequencing approaches.

Somatic mosaicism has been reported as an etiology of multiple IEIs, starting in the mid-2000s (Holzelova et al., 2004; Saito et al., 2005). Autoinflammatory and lymphoproliferative disorders are well represented among IEIs with reported mosaicism and offer key insights. The first example involves *NLRP3* (NOD-like receptor proteins 3), which encodes a protein involved in inflammasomes and, ultimately, the inflammatory response. Defects in this gene cause aberrant inflammation and a syndrome called NOMID (neonatal-onset multisystem inflammatory disease), which is characterized by persistent inflammation and tissue damage primarily affecting the nervous system, skin, and joints (Tanaka et al., 2011; Kuemmerle-Deschner et al., 2017; Nishikomori et al., 2019). Compared to individuals with germline *NLRP3* pathogenic variants, individuals who have somatic *NLRP3* variants restricted to the lymphoid and/or myeloid lineages are reported to have a notable lessening of the neurological impairment classically seen in NOMID. As an extreme example of this concept, a patient was reported with a recurrent urticaria-like rash, fever, conjunctivitis, and oligoarthritis in the 6^th^ decade of life. Genetic studies identified a novel pathogenic *NLRP3* variant with a low allele fraction, restricted to the myeloid lineage (Mensa-Vilaro et al., 2016). This case has provocative implications for the potential genetic contributions to other late-onset suspected IEIs as a future area of research and builds on related complex autoinflammatory disease findings (Hoffman and Broderick, 2017).

Other IEIs with somatic mosaicism as a reported etiology include but are not limited to *FAS*-related autoimmune lymphoproliferative syndrome (Dowdell et al., 2010); *TLR8*-related immunodeficiency with bone marrow failure (Aluri et al., 2021); and *UBA1*-related severe and often fatal, adult-onset autoinflammatory disease (Beck et al., 2020). For some of these disorders, the constitutional presence of the pathogenic variant is yet to be reported and may be incompatible with life (e.g., *UBA1*), while for others somatic mosaicism is a contributing etiology alongside inherited variants (e.g., *FAS*). Timing of the somatic event is paramount in determining the variant allele fraction and distribution of the variant among cells (Van Horebeek et al., 2019). We anticipate more somatic variants causing IEIs at older ages will be identified in the coming years.

Apart from somatic mutations directly causing disease, acquired, somatic variants may also occur as part of the progressive development of an important underlying disease entity, as is seen in GATA2 deficiency-related myeloid transformation (West et al., 2014; McReynolds et al., 2019; West et al., 2022). *GATA2* (GATA binding protein 2) encodes a transcription factor involved in regulating the transcription of genes involved in the development and proliferation of hematopoietic cell lineages (Spinner et al., 2014). Defects in this gene cause a multi-system syndrome encompassing immunodeficiency, myelodysplastic syndrome/acute myeloid leukemia, pulmonary disease, and vascular/lymphatic dysfunction (Spinner et al., 2014; Donadieu et al., 2018). Phenotypic variability observed in these families is striking and can pose clinical challenges regarding the ideal timing of HSCT (Donadieu et al., 2018). By better understanding the cumulative somatic events that foretell myeloid transformation, HSCT timing can be more effectively tailored which can be monitored by NGS-based monitoring with MDS/AML panels, similar to current follow-up of *ELANE* related severe congenital neutropenia or Shwachman Diamond syndrome with a cumulative risk of clonal myeloid transformation (Beck et al., 2020). Similarly, for families with heterozygous pathogenic variants in *FAS*, incomplete penetrance of lymphoproliferation and autoimmunity can be partially explained by somatic changes in the second *FAS* allele in the lymphoid compartment (Magerus-Chatinet et al., 2011), consistent with models of multistep pathogenesis for malignant transformation (Goodnow, 2007).

As a remarkable point of contrast to these relatively precise somatic changes, chromothripsis, the catastrophic cellular event in which chromosomes undergo massive deletion and rearrangement, has been reported as a spontaneous cure for WHIM syndrome (McDermott et al., 2015). WHIM syndrome is caused by defects in the gene *CXCR4* and is characterized by neutropenia, B cell lymphopenia, myelokathexis, hypogammaglobulinemia, recurrent infections, and marked susceptibility to human papillomavirus infection with resultant warts (Kawai and Malech, 2009). In the unique case report, the somatic, chromothriptic event deleted the pathogenic *CXCR4* allele and other genes in a hematopoietic stem cell. This led to the spontaneous and durable clinical remission of the patient’s long-standing warts, neutropenia, and myelokathexis in her 4^th^ decade of life (McDermott et al., 2015).

Complementing the insights from the compelling clinical course for this patient with WHIM syndrome, somatic reversion has long been described in the setting of IEI and refers to the spontaneous repair of a germline defect, resulting in improvement of the mutated molecular phenotype in progeny cells (Pillay et al., 2021). Over a dozen diseases including but not limited to those caused by defects in *DOCK8*, *WAS*, *ADA*, *RAG1*, and others have reports of somatic reversion (Miyazawa and Wada, 2021). Such reversion mutations can offer a significant proliferative or survival advantage to affected cells, thus altering the disease course (Humblet-Baron et al., 2007).

The fundamental importance of somatic mosaicisms in explaining phenotype variation within IEIs is underscored by these contrasting examples of somatic events accelerating and ameliorating disease. As a field, we are pivoting our understanding of the genetic contribution to disease from that of a limited, static inheritance to a more sophisticated and accurate model of each individual as a dynamic, complex mosaic of genetically distinct cells that change over time.

#### 2.1.3 Allelic bias

Allelic bias in gene expression is another mechanism that holds key lessons for understanding biology, and potentially, phenotypic variation. Monoallelic expression is an extreme form of allelic bias. Monoallelically expressed genes generally belong to one of three groups: (1.) parent-of-origin imprinted genes, where all cells have the same active allele that is epigenetically determined solely by the allele’s parent-of-origin; many of these genes govern early growth and development and will not be discussed here; (2.) random X-inactivation, and (3.) random allelic bias among autosomal genes (Chess, 2016).

X-inactivation occurring early in female embryonic development is a well-established dosage compensation mechanism ensuring that X-chromosome genes are expressed at comparable levels in males and females. Stochastic deviation from a 50/50 distribution of X inactivation is common (Shvetsova et al., 2019). Inactivation is hypothesized to be further skewed in the presence of negative selection. For example, chromosomal aberrations on the other allele are known to promote skewed inactivation, as is monozygotic twinning. When this X-inactivation is highly skewed toward the expression of an X-chromosome containing a pathogenic variant, females can develop symptoms typically observed in males with the X-linked disease; however, skewed X-inactivation does not seem to explain all cases of manifesting carriers (Migeon, 2020). See **Figure 2D** for illustration.

Within IEI, this phenomenon is best described in *CYBB*-related chronic granulomatous disease (CGD). *CYBB* (gp91*^phox^*) encodes a major protein of the NADPH oxidase complex, which plays a critical role in the destruction of pathogens by phagocytes. *CYBB*-related CGD is characterized by recurrent life-threatening bacterial and fungal infections. In a study of 93 CGD carriers, skewed X-inactivation was associated with infection risk. Interestingly, the carrier state itself, regardless of the balance of X-inactivation, was associated with autoimmunity (Marciano et al., 2018). Smaller cohort studies and case reports replicate this finding (Rupec et al., 2000; Cale et al., 2007; López-Hernández et al., 2019). It is worth noting that female carriers of *CYBB*-related CGD can exceptionally develop manifestations of CGD, as described in a recent case report of successful HSCT for a female carrier with severe life-threatening, late-onset granulomatous colitis and recurrent lung infections (Trevisan et al., 2022).

Concurrent with the discovery of the mechanisms governing random X-inactivation in the 1960s, random monoallelic expression was described in the autosomal genes encoding antigen receptors in T and B lymphocytes (Jack and Du Pasquier, 2019). For each lymphocyte, only one of the two alleles of the antigen receptor is expressed. To accomplish this, somatic V(D)J rearrangement is completed first on one of the two alleles present. Additional rearrangement is inhibited when a functional receptor is expressed on the surface of the cell (Rada and Ferguson-Smith, 2002; Jack and Du Pasquier, 2019). In subsequent decades, other autosomal genes were found to be subject to random monoallelic expression including, interleukin genes (i.e., *IL2*, *IL3*, *IL4*, *IL5*, *IL10*, *IL13*), and NK cell receptor genes (i.e., *KIR* genes) (Kelly and Locksley, 2000; Chan et al., 2003; Calado et al., 2006). Further work identified widespread random monoallelic expression affecting more than 5% of assessed genes (Gimelbrant et al., 2007; Chess, 2016). This random monoallelic expression increases the potential for cellular diversity within tissues because it creates up to three distinct cellular states (i.e., cells expressing a gene biallelically or from either of the two alleles) (Chess, 2016).

There are limited examples linking autosomal monoallelic expression to clinical disease in immunology. A recent case report describes a patient with early-onset multi-organ immune dysregulation resulting from a somatic pathogenic variant in *JAK1. JAK1* (Janus kinase 1) is a critical player in immune signal transduction. In the reported case, the authors demonstrated that *JAK1* was monoallelically expressed (i.e., only the mutated allele was expressed) across multiple immune cell subsets and not in other tissue cells as control (Gruber et al., 2020). When the cellular diversity created by allelic bias is considered in combination with the critical developmental windows in immune maturation, it is possible that such monoallelic expression of immune genes in hematopoietic or non-hematopoietic cells contributes to phenotypic variation in autosomal dominant diseases and may prove to be an important point of focus for future studies of penetrance (Gui et al., 2017).

#### 2.1.4 Multi-locus genetic variation

A final genetic contribution for intra- and interfamilial phenotypic variation for IEI is multi-locus genetic variation. In its simplest form, phenotypic outliers can occur when an individual has multiple genetic disorders. Studies of “dual diagnosis” show that it is more common for co-occurring genetic disorders to affect distinct rather than overlapping organ systems (Posey et al., 2017; Similuk et al., 2022). The co-occurrence of two or more IEIs in one individual is a statistically rare event, although several such cases have been reported (Chinn et al., 2017; Rae et al., 2018). Moderate penetrance alleles maintained at slightly higher population frequencies, such as pathogenic *TNFRSF13B* variants, have been reported in multiple families (ThaventhIran et al., 2020; Hargreaves et al., 2022; Similuk et al., 2022). Such cases are suggested to be more likely to manifest evidence of autoimmunity, lymphoproliferation, and/or common variable immunodeficiency over time than would otherwise be seen (Salzer and Grimbacher, 2021). See **Figure 2E** for illustration.

In addition, increasing evidence of mitigated phenotypes in carriers of recessive Mendelian disorders requires us to reconsider what we understood as settled science. A few early studies provided clues to this phenomenon, despite challenges with statistical power and ascertainment bias (Chillón et al., 1995; Girodon et al., 1997). Subsequent SNP-array-based genotyping studies were limited in their sensitivity for detecting rare disease variants for investigation. Only with the current generation of population biobank cohorts can this phenomenon begin to be clarified among a broader set of phenotypes and a wider range of recessive disorders (Barton et al., 2022). Although not an IEI, *CFTR*-related cystic fibrosis provides a phenotypically-relevant example where *CFTR* carriers have been shown in multiple studies to be at increased risk of cystic fibrosis-associated phenotypes including asthma, aspergillosis, and bronchiectasis (Çolak et al., 2020; Miller et al., 2020; Barton et al., 2022). For other phenotypically relevant examples: heterozygous carriers of a disease variant in *ABCA3* (associated with autosomal recessive interstitial lung disease) show evidence of decreased lung function and heterozygous carriers of a missense variant in *TG* (associated with autosomal recessive thyroid dyshormonogenesis) show an increased hypothyroidism risk (Barton et al., 2022). Thus, carrier status, long relegated to only being relevant for reproduction, may be relevant in certain circumstances to both complex disorders and individual susceptibility over time. It is yet to be determined the extent to which such information will be integrated into clinical practice, along with the most effective ways to communicate complex, multi-locus risk information. Nonetheless, it is likely that the coming years will further illuminate the spectrum of variant effects in immune defects as demonstrated for *TNFRSF13B* (TACI) or *MBL2* encoding mannose-binding lectin as disease-modifying variants of relevance, including mitigated phenotypes among carriers of recessive gene variants, along with the relevant mechanisms of action.

Digenic inheritance is often described as the simplest form of complex inheritance, although it classically requires the two implicated genes to have interacting gene products, leading to few examples. Within IEI, the best-supported example involves 6 autoinflammatory patients from 4 families with heterozygous pathogenic missense variants in an inducible proteasome subunit (*PSMB8* or *PSMB9*) and a heterozygous pathogenic variant in a constitutive proteasome subunit (*PSMB4* or *PSMA3*). The proteins encoded by these genes form a proteasome complex, which helps maintain protein homeostasis and control inflammation (Torrelo, 2017). For these reported patients, functional evaluation demonstrated proteasome dysfunction, increased type I interferon production, and, subsequently, chronic atypical neutrophilic dermatosis with lipodystrophy and elevated temperature (Brehm et al., 2015).

Considerably more complex and without current clinical applications in immunology, polygenic risk modeling is another rapidly evolving technology that may explain aspects of inter- and intra-familial phenotypic variation within IEI. Notably, atopy, autoimmunity, and autoinflammation are highly heritable conditions with partially overlapping susceptibility loci, despite potentially counteracting immune mechanisms (Kreiner et al., 2017). For example, in a cohort of 1540 patients with systemic lupus erythematosus higher polygenic risk scores significantly correlated with earlier age of diagnosis (Dominguez et al., 2021). Additionally, in a large, multi-ethnic cohort analyses identified *population-specific* effects of common and rare variants associated with inflammatory bowel disease (Gettler et al., 2021). In the future, it may be possible to stratify IEI penetrance via common variant contributions, often in genes known to also explain IEI. Additional controversy exists regarding the extent to which polygenic risk models will be able to capture a meaningful proportion of variation in risk or translate clinically from the cohort to an individual patient (Wand et al., 2021). The integration of variant effects across the frequency spectrum, and in a manner that does not assume identical genetic architecture across ancestral populations, is a rapidly evolving area of investigation.

### 2.2 Environment

Our discussion of environmental influences on phenotypic variation in IEI will begin with a review of the hygiene hypothesis, a classic body of work in disease epidemiology. This will be followed by contemporary discussions of microbial dysbiosis, hormonal influences, and specific pathogens as triggers.

#### 2.2.1 Early life infectious burden

Much of our understanding of the environmental factors shaping risk for immune-mediated disease can be broadly summarized in the hygiene hypothesis, which postulates that the overall decreased infectious burden over the last several decades has contributed directly to a rise in atopic and autoimmune disease (Garn et al., 2021). It should be emphasized that despite its misnomer, socioeconomic development is more relevant to these trends than one’s hygiene habits. This pattern was first described in the context of atopy more than 30 years ago, has been well supported by epidemiological and experimental data, and has been remarkably adaptable to emerging science (Bach, 2018). While it seems clear that early life infectious burden has a protective effect against immune-mediated diseases across the lifespan, the exact infections, disease-specific developmental windows, and underlying mechanisms are multiple, complex, and not well understood (Bach, 2002; Bach, 2018; Bach, 2020; Garn et al., 2021). The extent to which these patterns influence individuals or families with IEI is unknown.

Other environmental exposures have also been linked to the rise in atopic and autoimmune disorders in the industrialized world, such as smoking, chemical exposures, and air pollution, which likely affect risk via multiple mechanisms including inflammation (Costenbader and Karlson, 2006; Kantor and Silverberg, 2017; Zhao et al., 2019; Luschkova et al., 2021; Woo et al., 2022).

#### 2.2.2 Microbial dysbiosis

The microbiome’s dynamic, bidirectional role in influencing immune system development and function and wide-ranging disease pathogenesis has also garnered great interest. The genomics advances of the 21^st^ century have made it possible to characterize microbiome diversity and composition to an extent not previously possible. Westernized diets (high in fat and simple sugars), broad-spectrum antibiotic use, and other early-life exposures (largely encompassed by the hygiene hypothesis) are all implicated in microbial dysbiosis (Dethlefsen and Relman, 2011; Maurice et al., 2013; Modi et al., 2014; Sonnenburg et al., 2016; Gilbert et al., 2018; Hills et al., 2019). Microbiome composition is uniquely adapted for local body sites and is best understood in the gut. Gut dysbiosis is typically characterized by a decrease in microbiota diversity, with a decrease in specific commensals and an increase in harmful bacteria. While dysbiosis is associated with numerous immune disorders including inflammatory bowel disease, type 1 diabetes, multiple sclerosis, lupus, atopic dermatitis, and allergy, the extent to which dysbiosis plays a role in triggering disease, disease progression, and/or is, in fact, a consequence of the disease itself is unclear to date (Kostic et al., 2015; Bach, 2018; Liu et al., 2022; Xiang et al., 2022). While we are only beginning to understand the complexities involved, current research seems to be pivoting from descriptive and early experimental studies to a more mechanistic and translational emphasis (Gilbert et al., 2018). There is substantial enthusiasm for the therapeutic potential of modulating the microbiome. As a first-in-kind therapy, the FDA approved microbiome transfer for recurrent and refractory *Clostridium difficile* infection. Many other interventions are under investigation (Hendricks et al., 2019; Waller et al., 2022).

Multiple studies have sought to characterize the microbiome in individuals with IEI and related animal models, as either the cause and/or result of immune dysregulation. A unifying finding has been the depletion of intestinal commensals (Pellicciotta et al., 2019). Despite tremendous challenges, investigators have been eager to translate findings from common, complex disease into the setting of IEI, as well as contribute lessons from the in-depth study of rare disease to common, complex diseases (Pellicciotta et al., 2019; Al-Nesf et al., 2021).

#### 2.2.3 Internal hormonal milieu

Another fundamental vector shaping immunity and disease risk is sex. Differences in male and female immunity are well documented, but not well understood. Generally, females have a more robust immune response as evidenced in response to vaccination, sepsis, and certain other infections, including COVID-19 (Flanagan et al., 2017; Patin et al., 2018; Gupta et al., 2020). Inversely, female sex is a risk factor for multiple autoimmune and inflammatory disorders (Ngo et al., 2014; Billi et al., 2019; Natri et al., 2019; Ortona et al., 2019). Analogous to other environmental factors discussed in this review, sex and gender undoubtedly affect immunity and disease risk through multiple mechanisms. Some mechanisms are likely to be hormonally driven, as has been supported by comparisons in innate immunity amongst healthy controls, subjects with Klinefelter syndrome (XXY) (Holzelova et al., 2004), and pre-pubescent children (Gupta et al., 2020); other mechanisms may be directly linked to genetic factors differentiating males and females, such as those related to X-inactivation; and finally, other effects may be entangled in social lifestyle differences across genders (Billi et al., 2019).

It is not clear if or how the sex- or gender-related trends described above manifest across IEIs or within specific IEIs (Sheikhbahaei et al., 2016; Abolhassani et al., 2020), although the question is complex and the possible mechanisms of effect would likely differentially impact specific IEIs. More work in this area is needed.

#### 2.2.4 Pathogens as triggers

A final and more concrete discussion point amongst environmental factors shaping phenotype variation in IE is the role of pathogens as disease triggers. As a foundational concept, specific viral exposures, most notably cytomegalovirus (CMV), are well appreciated for their broad influence on immune variation as compellingly demonstrated by studies of healthy controls and monozygotic twins (Kuijpers et al., 2003; Brodin et al., 2015; Patin et al., 2018). However, in the setting of an IEI, a viral infection such as CMV, which is typically latent, can profoundly catalyze disease processes with fatal consequences. CMV may be encountered early in life being a frequent viral infection transmitted shortly after birth by breastfeeding (Ruffner et al., 2017; Drutman et al., 2020; Chong-Neto et al., 2022). Also, Epstein-Barr virus (EBV) provides a similar example. Like CMV, EBV is a herpesvirus that latently infects much of the global population and infects predominantly B cells. Multiple IEIs exhibit a striking and specific susceptibility to EBV-related diseases such as mononucleosis, lymphoproliferative disease, hemophagocytic lymphohistiocytosis, and/or EBV+ lymphoma. Notably, individuals may be completely asymptomatic or have a mild clinical course before EBV infection triggers a dramatic progression of disease; examples include disorders related to the genes *SH2D1A*, *XIAP*, *ITK*, *MAGT1*, *CD27*, *CD70*, among others (Tangye et al., 2017; Tangye and Latour, 2020). Other examples of specific viral susceptibility include autosomal recessive OX40-deficiency and human herpes virus 8, leading to exceedingly rare childhood-onset Kaposi sarcoma, as well as, possibly, the recently described TLR7-deficiency leading to aberrant recognition of ssRNA viruses, decreased interferon response to SARS-CoV-2 infection, and increased risk of life-threatening COVID pneumonia (Byun et al., 2013; Asano et al., 2021).

Even for IEIs which are not defined by a severe susceptibility to viral disease, CMV or EBV infection may significantly change the disease course by inducing lymphoproliferation or other complications, as has been observed in RAG-deficiencies, CTLA4-haploinsufficiency, activating PIK3∂ syndrome, and other IEIs (Schwab et al., 2018; Edwards et al., 2019; Bosticardo et al., 2021; Thouenon et al., 2021). Additionally, it was relatively recently discovered that the persistence of vaccine-derived rubella virus appears to be a crucial, triggering factor for many cutaneous and visceral granulomas in individuals with IEI, a phenotype whose only effective management to date is HSCT (Perelygina et al., 2020; Perelygina et al., 2021; Notarangelo, 2022).

There are numerous other examples of pathogens as dramatic triggers of IEI phenotypes, including in otherwise healthy-appearing individuals. BCG vaccination of individuals with MSMD offers a particularly discrete example involving a bacterial pathogen (Fekrvand et al., 2020). Live attenuated virus vaccinations have also been known to reveal previously undiagnosed IEI, with particularly severe consequences in individuals with SCID, as well as individuals with innate immune defects (e.g., *STAT2*, *IRF9*) (Gothe et al., 2021). Likewise, CARD9 deficiency underlies invasive fungal infections, primarily reported in individuals who were apparently healthy before the precipitation of their severe, and in some cases, fatal infections (Drewniak et al., 2013; Rieber et al., 2016; Corvilain et al., 2018; Imanaka et al., 2021). Within the broader epidemiological trends increasing the prevalence of common, complex immune disorders across industrialized populations, exposure to specific pathogens remains a serious risk factor for triggering disease in individuals with IEI.

### 2.3 Time

In the frequently referenced paradigm of gene-environment interplay, time is an often missed “third factor,” which we now highlight (Boyce et al., 2020). As a straightforward interpretation of time’s role in phenotypic variation, one can consider the longitudinal unfolding of a disease’s natural history for a given individual over time. While many IEIs have a fundamentally progressive disease course, exceptions do exist: MYD88 and IRAK4 deficiencies cause early-onset life-threatening infection with a small number of bacterial species, the severity and frequency of which lessen over time, even with decreased reliance on prophylactic antibiotics (von Bernuth et al., 2012). A closely related consideration is time’s role across an individual’s lifecycle: implicating the dynamic, programmed modulation of immune function as an individual progresses from the neonatal period to adulthood, including possible pregnancy, and into later life (Simon et al., 2015; Patin et al., 2018; Natri et al., 2019). Critical developmental windows exist for immune system maturation, particularly in the neonatal period, the disruption of which may have lifelong effects (Al Nabhani and Eberl, 2020). From an alternative and similarly concrete perspective, timing plays a central role in some of the mechanisms discussed above, such as somatic variation where the timing of the somatic event(s) largely determines key characteristics such as somatic variant allele fraction and distribution of the somatic variant across the soma, or even acceleration or amelioration of disease.

The timing of exposure to pathogens and subsequent intervention are deeply consequential for a given individual, as described in the case report of an asymptomatic, EBV-negative young adult male with XIAP deficiency (identified by family history) who required extensive treatment for depression in adjusting to living at-risk of a life-threatening disorder with a ubiquitous viral trigger (Durkee-Shock et al., 2021). These issues of timing are not routinely studied systematically in clinical immunology, suggesting avenues for future research, as well as relevant clinical counseling issues (Brodin, 2020).

## 3 Discussion

In this review, we have provided an updated summary of key concepts in genetic and environmental contributions to phenotypic variation within IEI. For genetics, this has included differential variant impact, somatic events, allelic bias, and multi-locus genetic contributions. From the environmental perspective, we’ve covered the epidemiological impact of decreased infectious burden over recent generations, microbial dysbiosis, hormonal influences, and pathogens as critical triggers for individuals with IEI. Our review may be limited by the inadvertent omission of relevant literature or missed connections. Although the above factors have been reviewed separately, their impact on phenotype is intrinsically one of reciprocal interplay and is dynamic over time. Reductionist approaches have successfully generated detailed insight into many of the building blocks of biological systems and can fail to capture this dynamic, complex nature.

Across science, we tend to overestimate innovation in the short term but underestimate its impact in the long term. The recent history of genetics is exemplary in this regard: the decade following the completion of the human genome project saw minimal translation into clinical care and certainly not a transformation of medicine. And yet, the early genetic research of this century gave rise to innovations in methods, new applications, and fundamental learning in genome biology. This same period has seen the identification of almost 500 causes of IEI, as well as major advances in our ability to characterize somatic events, the microbiome, and genotypes across large populations. Although the progress has not been linear, these developments have accumulated into an enhanced ability to diagnose and treat IEI, in some cases with effective precision therapy or definitive interventions like HSCT. And yet, anyone who has sat with a family affected by IEI as they grapple with the uncertain prognosis of their disease knows the limits of this progress. The substantial challenge looking forward is to organize information in such a way that accommodates the genotypic and phenotypic heterogeneity within IEI and integrates this information to arrive at a more comprehensive and accurate understanding of how the immune system operates in health and disease (Germain et al., 2011; Germain, 2018).

## Author contributions

MS and TK provided joint contributions to the conception and design of the work, drafting the work and revising it critically for important intellectual content. Both authors also provide approval for publication of the content and agree to be accountable for all aspects of the work in ensuring that questions related to the accuracy or integrity of any part of the work are appropriately investigated and resolved.
